# Plasmacytoma of the skull mimicking an epidural hematoma: A case report

**DOI:** 10.1097/MD.0000000000015443

**Published:** 2019-04-26

**Authors:** Zupeng Chen, Xu Li

**Affiliations:** Department of Neurosurgery, The First Affiliated Hospital of Zhejiang Chinese Medical University, Hangzhou, Zhejiang Province, China.

**Keywords:** epidural hematoma, multiple myeloma, plasmacytoma, skull tumor

## Abstract

**Rationale:**

Plasmacytoma as the 1st presentation of skull tumors is a rare disorder. When it is combined with brain trauma or dramatic changes in intracranial pressure, patients are more prone to misdiagnosis.

**Patient concerns:**

A 67-year-old woman complaining of a headache presented with a history of head trauma for the past 1 hour. Emergency head computed tomography initially suggested an epidural hematoma.

**Diagnosis:**

Emergency surgery was performed to remove the intracranial hematoma, but a tumor-like mass was found during surgery, and pathologic assessment confirmed plasmacytoma. Surgery was difficult because of bleeding. The tumor was radically removed.

**Interventions and outcomes:**

The patient underwent whole-brain radiotherapy and chemotherapy. She died 40 months after the surgery.

**Lessons:**

Epidural lesions found after a head injury may be assumed to be an epidural hematoma, leading to unnecessary surgery. Diseases such as hematomas, meningiomas, eosinophilic granulomas, bone metastases, and osteosarcomas must be considered.

## 1 Introduction

Multiple myeloma is a neoplastic lesion that usually originates in bone marrow, including that from the pelvis, skull, vertebrae, and ribs. Multiple myeloma constitutes approximately 1% of all malignant neoplasms. The disease may also have very rare extraosseous manifestations, which can arise in any tissue, but are found in <5% of patients with multiple myeloma.^[1]^ Skull plasmacytomas have characteristic features including scattered, round, chisel-like, or oil-like bone defects; lesions of different sizes detected using skull X-ray; and clinically difficult to diagnose, resulting in misdiagnosis. However, skull plasmacytomas initially showing mass-like tumors are rare in clinical practice and are easily misdiagnosed as meningioma or metastatic tumors.^[1–3]^ We report a patient with a skull plasmacytoma and discuss the preoperative clinical diagnosis of this patient. There was no systemic involvement in this case.

## 2 Case report

**Figure 1:**
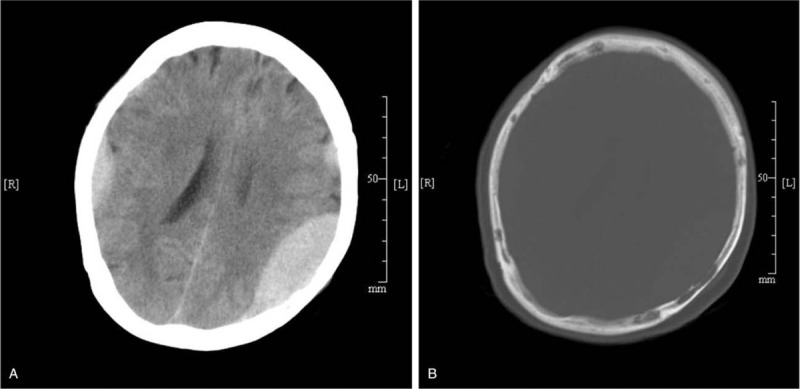
(A) Computed tomography revealed a 6 × 3 × 6.5-cm, epidural, slightly dense mass in the left parietal region and 2 smaller epidural masses in other regions. The left mass was compressing part of the brain, the left ventricle was smaller than the right ventricle, and the midline was not shifted. (B) The skull showed patchy osteolysis, and there were small lytic changes.

In November 2014, a 67-year-old woman was admitted to our hospital because of head trauma. She had a history of a headache for 2 weeks before the trauma. On physical examination, a firm, nontender, subcutaneous nodule 2 cm in diameter was observed in the parietal region. Her Glasgow coma scale (GCS) score was 14. Computed tomography (CT) of the head revealed a 6 × 3 × 6.5-cm, epidural, slightly dense mass in the left temporoparietal region and 2 smaller epidural masses in other regions. The left mass was compressing part of the brain but had well demarcated to the dura. There was no evidence of a midline shift, but the left ventricle was smaller than the right ventricle (Fig. 1A). The skull showed patchy osteolysis, and there were small lytic changes (Fig. 1B). Because of the history of head trauma, the tumor was not regarded as a plasmacytoma, and a radiologic diagnosis of typical epidural hematoma was suggested.

**Figure 2:**
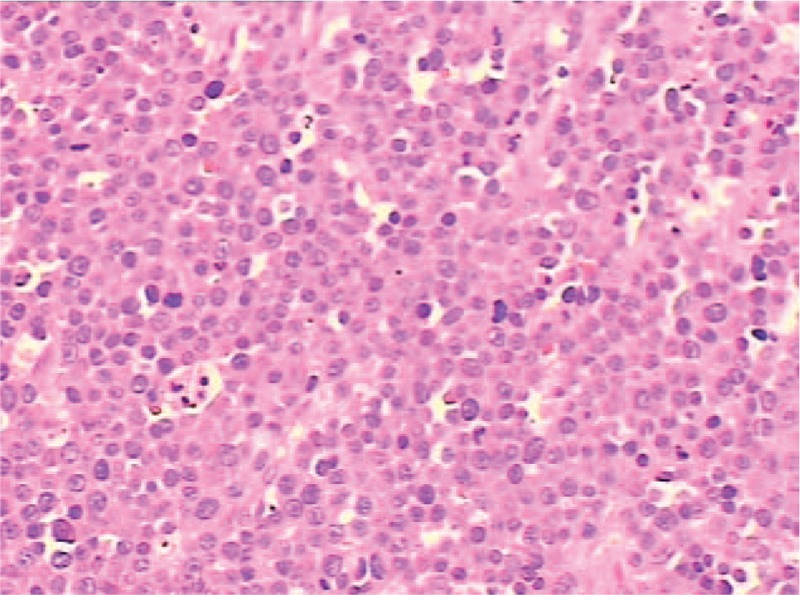
Hematoxylin and eosin staining showed that the tumor was composed of plasma cells throughout the field (×200).

After admission, the patient's condition deteriorated rapidly. Her GCS score dropped to 12 in 2 hours, there were signs of increased intracranial pressure, she was disoriented, and she developed right hemiplegia. Her neurologic status was not stable. An immediate surgery was scheduled. The patient underwent left fronto-temporo-parietal craniectomy. At this point, significant skull bleeding was observed, and hemostasis was very difficult. We quickly removed the skull and used bone wax to manage the bone window edge. During the surgery, a gray-red tumor was found to have infiltrated the left temporo-parietal bone, there was no dura defect, and the margin between the tumor and brain parenchyma was regular. The mass was excised, and the dural surface adherent to the mass was coagulated. Histologic examination revealed a plasmacytoma of the bone with extensive amyloidosis; the tumor tissue consisted of sheets of immature plasmacytic cells (Fig. 2).

The patient underwent a series of postoperative evaluations; her urine Bence Jones protein test result was negative, serum calcium level was 3.34 mmol/L, and plasma electrophoresis for immunoglobulin A increased slightly. The complete blood count and complete metabolic profile showed no abnormalities, although her renal function remained unimpaired. The patient underwent bone marrow aspiration, the results of which were also negative.

Surgical resection was followed by adjunctive 5400-cGy radiation therapy of the cranial lesion with the knowledge that a plasmacytoma is a radiosensitive tumor. She received 6 cycles of chemotherapy containing a bortezomib, melphalan, and prednisone regimen after the radiotherapy. On follow-up examination 12 months later, no evidence of multiple myeloma was detected, but a pathologic fracture of the left humerus was observed on follow-up at 25 months, and she died of pulmonary infection 40 months later.

## 3 Discussion

A plasmacytoma is a cancer that originates from precursors of plasma cells (pre-B cells). The intracranial manifestations of plasmacytomas as mass lesions are rare and easily misdiagnosed. Cranial tumors may also be the only manifestation of plasmacytomas. Moulopoulos et al found that pure central nervous system involvement without any bone lesions is very rare, occurring in <1% of patients with multiple myeloma.^[4]^ Luigi and Maurizio reported that a right fronto-parietal skull plasmacytoma was found on CT; the patient underwent total removal of the tumor by craniotomy was not followed by any chemotherapy or radiotherapy, and never developed systemic neoplastic disease in 3 years.^[5]^ In contrast, Kyle stated that >80% of patients with an apparently solitary plasmacytoma will eventually develop myeloma.^[6]^

Patients with solitary skull plasmacytomas often present with several months of slight headaches^[5,7]^ or hearing disturbances,^[8]^ while some patients might have no symptoms at all. Nguyen and Patel noted that a plasmacytoma in one patient was found by her hairdresser.^[9]^ However, in our case, the patient had a short history of a headache, and mixed with head trauma, these circumstances confused the doctor's judgment. CT showed that a destructive or well-demarcated soft-tissue mass was compressing part of the brain. On magnetic resonance imaging, the tumor was isointense relative to the brain and enhanced on T1-weighted imaging.^[5,7,9]^ Unlike multiple myeloma, solitary skull plasmacytomas may not have serum M protein or Bence Jones light chains in the urine.^[3,5,7,9]^ The diagnosis is usually made after pathologic tissue is obtained after surgery. Examination of a bone-marrow biopsy specimen is needed.^[3,7,9,10]^

The tumor may exhibit invasion from the subcutaneous tissue to the brain parenchyma or from the dura mater to the brain.^[11]^ The dura is involved in plasmacytoma infiltration in some patients with these diseases. Solitary intracranial plasmacytomas without any other signs of multiple myeloma have been associated with a good prognosis after surgical or radiation therapy^[3,7,9]^; on the contrary, the development of extraosseous manifestations in multiple myeloma are characterized by rapid progression and resistance to treatment.^[12]^ Some authors consider that it is best to administer radiotherapy after histologic diagnosis of the tumor, as this tumor is highly radiosensitive.^[7,8]^ Mäntylä et al and Yang et al have argued that gross total removal of the tumor followed by chemotherapy is successful.^[2,13]^ Nguyen and Patel stated that a patient had undergone autologous bone marrow transplantation after the mass was excised.^[9]^ Alessandro et al suggested that combined therapy (chemotherapy followed by radiotherapy after surgery) can be very efficacious.^[10]^

Plasmacytomas originating from the calvarium are usually rich in vasculature, and pathologic vessels and a tumor stain may be observed on angiography. In this case, the assessment was insufficient before surgery; the patient was not considered to have a plasmacytoma, and bleeding during the surgery lead to increased patient risk. When the preoperative diagnosis is a skull plasmacytoma, the neurosurgeon should pay attention to special control of the skull bleeding and choose different methods of craniotomy. The process of opening the skull should include the process of blocking the blood supply: drilling the skull out of the edge of the tumor, then biting but not milling the bone from the edge of the tumor in the normal skull, and sealing the edge of the bone with bone wax quickly to stop bleeding. This method can reduce the bleeding during a surgery effectively. The tumor must be completely removed; otherwise, it is easy to cause bleeding again.^[2]^

Singleton and Koerner demonstrated the diagnostic challenge associated with one of the most common pathologic entities in neurosurgical practice: extradural mass lesions.^[14]^ Intracranial plasmacytomas have some clinical features that can contribute to identifying other tumors, such as hematomas, meningiomas, eosinophilic granulomas, bone metastases, and osteosarcomas; these include the following: Age: Plasmacytoma usually occurs in patients >40 years; the median age of onset is 62 years, and only 2% to 3% of patients are younger than the age of 30 years.^[15]^ Eosinophilic granulomas are always observed in children and young individuals, and they mostly present as a single mass (85%). Radiologic signs: CT features of skull plasmacytomas include a mass of slightly high density and containing multiple osteolytic damage in the cranial diploë, which breaks through the cortical bone and erodes the surrounding soft tissue. Calcification can be observed in the mass; the minor bone involvement could be explained by pressure erosion; because of the pressure phenomenon, meningiomas may cause osteolysis, but this is usually associated with areas of sclerosis and thickening. Osteosarcomas and bone metastases also have osteolytic types, but the edge of the bone is blurred. Laboratory examination including bone marrow, serum protein electrophoresis, serum immunoglobulin, blood routine, Bence Jones protein, and renal function assessments. A systemic examination is necessary to evaluate the state of the body in some patients who have a solitary skull plasmacytoma. Solitary plasmacytomas are circumscribed neoplastic lesions with no clinical or radiologic signs of systemic involvement, accounting for about 7% of myelomas.^[16]^ In this way, usually nonspecific proteinuria and serum immunoglobulin develops, although renal function remains unimpaired.

## Author contributions

**Conceptualization:** Zu Peng Chen.

**Data curation:** Zu Peng Chen.

**Software:** Xu Li.

**Writing – original draft:** Zu Peng Chen, Xu Li.

**Writing – review & editing:** Zu Peng Chen.
